# Echocardiographic parameters of patients in the intensive care unit undergoing continuous renal replacement therapy

**DOI:** 10.1371/journal.pone.0209994

**Published:** 2019-01-11

**Authors:** Panagiotis Kompotiatis, Brandon M. Wiley, Jacob C. Jentzer, Kianoush B. Kashani

**Affiliations:** 1 Division of Nephrology and Hypertension, Department of Medicine, Mayo Clinic, Rochester, Minnesota, United States of America; 2 Department of Cardiovascular Medicine, Mayo Clinic, Rochester, Minnesota, United States of America; 3 Division of Pulmonary and Critical Care Medicine, Department of Medicine, Mayo Clinic, Rochester, Minnesota, United States of America; Scuola Superiore Sant'Anna, ITALY

## Abstract

**Main objectives:**

Echocardiographic parameters have been used to predict outcomes for specific intensive care unit (ICU) populations. We sought to define echocardiographic parameters for ICU patients receiving continuous renal replacement therapy (CRRT).

**Design, setting, participants, and measurements:**

This is a historical cohort study of consecutive ICU patients at Mayo Clinic (Rochester, Minnesota) who received CRRT from December 9, 2006, through November 13, 2015. Only patients with an echocardiographic examination within 7 days of CRRT initiation were considered.

**Results:**

The study included 1,276 patients. Decreased left ventricular ejection fraction (LVEF; ≤45%) was noted in 361/1,120 (32%) and increased right ventricular systolic pressure (RVSP; ≥40 mm Hg) was noted in 529/798 (66%). Right ventricular systolic dysfunction was observed in 320/820 (39%). The most common valvular abnormality was tricuspid regurgitation (244/1,276 [19%]). Stratification of these parameters by ICU type (medical, surgical, cardiothoracic, cardiac) showed that most echocardiographic abnormalities were significantly more prevalent among cardiac ICU patients: LVEF ≤45% (67/105 [64%]), RVSP ≥40 mm Hg (63/79 [80%]) and tricuspid regurgitation (50/130 [38%]). We compared patients with acute kidney injury (AKI) vs end-stage renal disease and showed that decreased LVEF (284/921 [31%] vs 78/201 [39%]), was significantly less prevalent among patients with AKI, but increased RVSP was more prevalent (445/651 [68%] vs 84/147 [57%]) with AKI.

**Conclusions:**

ICU patients who required CRRT had increased prevalence of pulmonary hypertension and right and left ventricular systolic dysfunction. Prediction of adverse outcomes with echocardiographic parameters in this patient population can lead to identification of modifiable risk factors.

## Introduction

Despite many advances in the care of critically ill patients and improvement in mortality rates during the past 20 years [1], the incidence of acute kidney injury (AKI) in patients in the intensive care unit (ICU) remains high (20%-57%) [2–4]. ICU patients with AKI have increased short- and long-term mortality rates (20%-27%) [3, 5], with the highest rates observed among patients with severe AKI who require acute initiation of renal replacement therapy. Of particular concern are patients whose hemodynamic instability necessitates initiation of continuous renal replacement therapy (CRRT). A 2015 meta-analysis reported an overall mortality rate of 46% for patients with AKI requiring CRRT [6], and individual studies have reported rates as high as 60% to 80% [7, 8].

CRRT was initially developed to treat patients who could not tolerate intermittent hemodialysis (IHD), either because of hemodynamic instability or because IHD could not efficiently correct acid-base and electrolyte disorders [9]. CRRT is also a practical tool in managing oligo-anuric critically patients with fluid overload or at risk of volume overload while requiring ongoing administration of parenteral drugs (e.g. vasopressors), blood products or parenteral nutrition [10, 11]. However, CRRT has not been proven superior to IHD in randomized control trials assessing mortality risk [12, 13]. Nevertheless, CRRT is often used in the ICU setting as a modality for renal replacement therapy, especially in clinical situations involving hemodynamic instability or intracranial hypertension, when IHD cannot be safely performed [13, 14].

Cardiac dysfunction, due to “cross-talk” between the heart and kidneys, is an important factor that often may contribute to development or worsening of AKI and progression to dialysis[15]. Echocardiography is the standard noninvasive method used to assess cardiac function in the ICU, and use of limited echocardiography to diagnose and guide therapy for patients with shock is associated with improved outcomes [16]. Further, the clinical relevance of various echocardiographic parameters has been described for patients in the ICU; these parameters include left ventricular ejection fraction (LVEF), diastolic function, right ventricle (RV) size, and tricuspid regurgitant jet velocity [17–19]. According to available evidence, RV size and tricuspid regurgitant jet velocity may be predictive of mortality risk [17, 18]. For patients receiving long-term hemodialysis, RV dysfunction is independently associated with increased mortality risk [20–22]. Pulmonary hypertension (assessed with echocardiography) also can indicate increased risk of death for patients with end-stage renal disease (ESRD) [21].

To our knowledge, the echocardiographic abnormalities in ICU patients receiving CRRT have not been characterized. We aimed to define the prevalence and patterns of echocardiographic abnormalities in ICU patients undergoing CRRT and to determine how these parameters differed among patients with AKI vs ESRD.

## Materials and methods

### Patient selection

The Mayo Clinic Institutional Review Board reviewed and approved this minimal-risk study with a waiver of informed consent (IRB # 15–008953). We retrospectively identified a historical cohort of all consecutive adults admitted to the ICUs at Mayo Clinic Hospital (Rochester, Minnesota), at the St. Marys and Methodist campuses, from December 9, 2006, through November 13, 2015. Patients were included in the study if they received CRRT during their ICU stay and had an echocardiogram within seven days (before or after) CRRT initiation. For individuals who had more than one echocardiogram performed within seven days of CRRT initiation, our analysis considered only the echocardiogram that was performed chronologically closest to the first CRRT session.

ESRD was defined per KDOQI[23] criteria as dialysis-dependent chronic kidney disease (CKD) stage 5 (Peritoneal or hemodialysis).

AKI was defined per KDIGO criteria based on both creatinine and urine output[14].

### Data collection

We reviewed electronic medical records and abstracted baseline patient characteristics. The severity of illness was determined by Acute Physiology and Chronic Health Evaluation (APACHE III) scores and Sequential Organ Failure Assessment (SOFA) scores. ICU data were obtained from an Epidemiology and Translational Research in Intensive Care ICU DataMart database [24, 25]. Comorbidities were assessed with the Charlson comorbidity index [26]. Data were collected using a standardized process (S1 File).

Echocardiographic parameters were extracted from a separate institutional echocardiography database. Mean LVEF is reported if it was calculated with more than 1 method. Left ventricular (LV) mass was measured with the linear method, and increased mass was defined as greater than 115 g/m^2^ for men and 95 g/m^2^ for women [27]. LV diastolic function was assessed by using the American Society of Echocardiography guidelines [28]. Left atrial volume was measured with a 2-dimensional method (reported as mL/m^2^). RV systolic dysfunction was defined as a moderate or greater decrease in RV systolic function (23), with RV systolic pressure (RVSP) estimated from tricuspid regurgitant jet velocity. Inferior vena cava (IVC) dilation was defined as IVC diameter greater than 2.1 cm [29]. Pericardial effusion was categorized as none, mild, moderate, or large [30]. Finally, valvular diseases (aortic, mitral, tricuspid) were assessed with color flow Doppler imaging and categorized as normal, mild, moderate, or severe stenosis or regurgitation [31, 32]. Data on valvular diseases were extracted as text from the echocardiographic reports; if no information about valvular disease was available, the valve was considered normal.

### Statistical analysis

Continuous variables are reported as mean (SD) or median (interquartile range [IQR]). Categorical variables are expressed as count (percent). Continuous variables were assessed by using the *t*-test, Wilcoxon rank sum test, analysis of variance, or Kruskal-Wallis test; categorical variables were assessed by using the χ^2^ test or Fisher exact test. JMP software (version 13.0.0; SAS Institute Inc.) was used for the analysis. *P* values < .05 were considered statistically significant.

## Results

**Fig 1:**
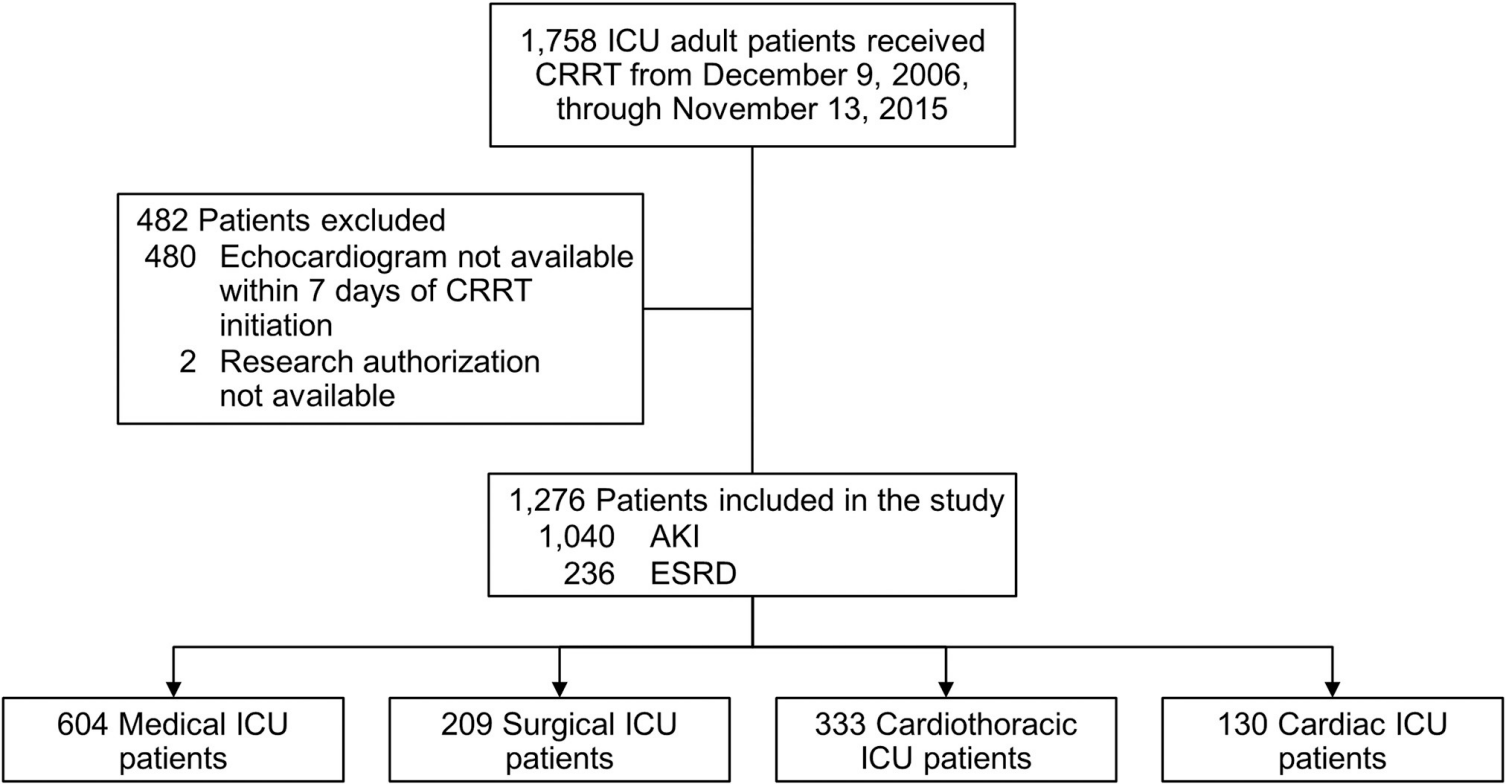
Patient enrollment flowchart.

During the study period, 1,758 unique patients received CRRT (Fig 1). We excluded 482 patients (27%); 480 did not have an echocardiogram within seven days of CRRT initiation, and 2 declined research authorization. The final analysis included 1,276 patients (Baseline characteristics, comorbid conditions, and outcomes are shown in Table 1. Among all enrolled patients, CRRT 82% of cases (n = 1,040) were due to AKI, and 18% of cases (n = 236) were provided to the ESRD patients. Regarding the CRRT modality, continuous venovenous hemofiltration was used in most cases (n = 1,268 [99%]), and slow continuous ultrafiltration was used for the other 8 patients (1%).

**Table 1:** Baseline characteristics, comorbidities, and illness severity (N = 1,276)^a^.

Characteristic	Value
Age, median (IQR), y	63 (53–73)
Female Sex, No. (%)	514 (40)
White Race, No. (%)	737/891 (83)
Weight, median (IQR), kg	87 (71–104)
Body Mass Index, median (IQR), kg/m^2^	29.30 (25.05–35.29)
Severity of Illness and Comorbidity, median (IQR)	
APACHE III score	99.5 (81–119)
SOFA score on the day of CRRT initiation	12 (10–14)
Charlson Comorbidity Index	5 (3–7)
Duration of CRRT, median (IQR), d	4 (2–9)
Indication for CRRT, No. (%)	
Acute Kidney Injury	1,040 (82)
End-Stage Renal Disease	236 (18)
Invasive mechanical ventilation during CRRT, No. (%)	937 (73)
Pulmonary Disease, No. (%)	207 (16)

Abbreviations: APACHE III, Acute Physiology And Chronic Health Evaluation; CRRT, continuous renal replacement therapy; IQR, interquartile range; SOFA, Sequential Organ Failure Assessment.

^a^ All patients received CRRT and had an echocardiogram available within seven days of CRRT initiation.

### Echocardiographic parameters

The median time between echocardiography and CRRT initiation was 8 hours (IQR, −10 to 31 hours). Echocardiographic findings of this cohort are summarized in Table 2. Identified abnormalities included IVC dilation, diastolic dysfunction, RV systolic dysfunction, and increased LV mass. The most common valvular abnormalities were tricuspid regurgitation and mitral regurgitation.

**Table 2:** Echocardiographic parameters.

Echocardiographic Parameters	Patients With Available Data, No. (%)	Value
LVEF, median (IQR), %	1,120 (88)	57 (39–65)
LVEF ≤45%, No. (%)	1,120 (88)	361 (32)
Diastolic Dysfunction, No. (%)	286 (22)	177 (62)
Increased Left Ventricular Mass, No. (%)	618 (48)	98 (16)
Left Atrial Volume Index, median (IQR), mL/m^2^	244 (19)	37 (29–49)
Right Ventricular Systolic Dysfunction	820 (64)	320 (39)
RVSP, median (IQR), mm Hg	798 (63)	45 (36–54)
RVSP ≥40 mm Hg, No. (%)	798 (63)	529 (66)
Inferior Vena Cava Dilation, No. (%)	804 (63)	573 (71)
Pericardial Effusion, moderate or greater, No. (%)	1,085 (85)	14 (1)
Aortic Regurgitation, moderate or greater, No. (%)	1,276 (100)	25 (2)
Aortic Stenosis, moderate or greater, No. (%)	1,276 (100)	19 (1)
Mitral Regurgitation, moderate or greater, No. (%)	1,276 (100)	100 (8)
Mitral Stenosis, moderate or greater, No. (%)	1,276 (100)	6 (<1)
Tricuspid Regurgitation, moderate or greater, No. (%)	1,276 (100)	244 (19)

Abbreviations: IQR, interquartile range; LVEF, left ventricular ejection fraction; RVSP, right ventricular systolic pressure.

Echocardiographic parameters, stratified by type of ICU admission (ie, medical, surgical, cardiothoracic, cardiac) are summarized in Table 3. The echocardiographic parameters that were statistically different between the different ICU types included median LVEF, diastolic dysfunction, IVC dilation, median RVSP, RV systolic dysfunction, moderate or greater aortic, mitral, and tricuspid regurgitation and left atrial volume index (Table 3). Lowest median LVEF, highest median RVSP and median left atrial volume index were noted in the cardiac ICU patients. IVC dilation, moderate or greater aortic, mitral, and tricuspid valves regurgitation were also more prevalent in cardiac ICU while RV systolic dysfunction was more prevalent in cardiothoracic surgery ICU.

**Table 3:** Echocardiographic data with statistical comparison between the different ICU types.

		ICU Type				
Echocardiography Parameter	Patients With Available Data, No. (%)	Medical	Surgical	Cardiothoracic	Cardiac	*P* Value
LVEF, median (IQR), %	1,120 (88)	60 (50–67)	55 (41–65)	50 (26–65)	36 (21–56)	< .001
LVEF ≤45%, No. (%)	1,120 (88)	125/566 (22)	58/185 (31)	111/264 (42)	67/105 (64)	< .001
Diastolic Dysfunction, No. (%)	286 (22)	97/177 (55)	27/50 (54)	26/30 (87)	27/29 (93)	< .001
Increased Left Ventricular Mass, No. (%)	618 (48)	53/373 (14)	18/98 (18)	21/97 (22)	6/50 (12)	.24
Left Atrial Volume Index, median (IQR), mL/m^2^	244 (19)	36 (27–45)	37 (31–51)	45 (31–52)	54 (41–64)	< .001
Right Ventricular Systolic Dysfunction, No. (%)	820 (64)	120/393 (31)	43/125 (34)	116/214 (54)	41/89 (46)	< .001
RVSP, median (IQR), mm Hg	798 (63)	46 (36–54)	45 (36–56)	44 (35–51)	49 (42–63)	.02
RVSP ≥40 mm Hg, No. (%)	798 (63)	292/443 (66)	83/131 (63)	91/146 (62)	63/79 (80)	.047
Inferior Vena Cava Dilation, No. (%)	804 (63)	312/453 (69)	74/123 (60)	120/158 (76)	67/71 (94)	< .001
Pericardial Effusion, moderate or greater, No. (%)	1,085 (85)	6/546 (1)	0/183 (0)	5/254 (2)	3/102 (3)	.09
Aortic Regurgitation, moderate or greater, No. (%)	1,276 (100)	3/604 (0.5)	2/209 (1)	14/333 (4)	6/130 (5)	< .001
Aortic Stenosis, moderate or greater, No. (%)	1,276 (100)	8/604 (1)	2/209 (1)	4/333 (1)	5/130 (4)	.18
Mitral Regurgitation, moderate or greater, No. (%)	1,276 (100)	29/604 (5)	7/209 (3)	31/333 (9)	33/130 (25)	< .001
Mitral Stenosis, moderate or greater, No. (%)	1,276 (100)	2/604 (0.33)	1/209 (0.48)	1/333 (0.30)	2/130 (1.54)	.31
Tricuspid Regurgitation, moderate or greater, No. (%)	1,276 (100)	105/604 (17)	32/209 (15)	57/333 (17)	50/130 (38)	< .001

Abbreviations: ICU, intensive care unit; IQR, interquartile range; LVEF, left ventricular ejection fraction; RVSP, right ventricular systolic pressure.

We next compared echocardiographic parameters between patients with AKI vs. ESRD (Table 4). Compared with patients with AKI, patients with ESRD had a lower median ejection fraction (54 vs. 75%, p = 0.04,) and a higher median left atrial volume index (43 vs. 36 mL/m^2^, p = 0.002). Additionally, a higher percentage of patients with ESRD had moderate or greater aortic (4 vs. 0.96%, p = 0.004) or mitral stenosis (2 vs.0.19%, p = 0.01), but a lower percentage of patients with ESRD had RVSP ≥40 mm Hg (57 vs. 68%, p = 0.009). The relationship between elevated RVSP and AKI development persisted even after adjusting for the presence of acute respiratory distress syndrome or mechanical ventilation.

**Table 4:** Comparison of echocardiographic parameters for patients with AKI vs. ESRD.

Echocardiography Parameter	No. (%)	Patients With AKI (n = 1,040)	Patients With ESRD (n = 236)	*P* Value
LVEF, median (IQR), %	1,120 (88)	57 (40–62)	54 (35–65)	.04
LVEF ≤45%, No. (%)	1,120 (88)	284/921 (31)	78/201 (39)	.03
Diastolic Dysfunction, No. (%)	286 (22)	134/226 (59)	43/60 (72)	.08
Increased Left Ventricular Mass, No. (%)	618 (48)	78/512 (15)	20/107 (19)	.37
Left Atrial Volume Index, median (IQR), mL/m^2^	244 (19)	36 (28–47)	43 (36–52)	.002
Right Ventricular Systolic Dysfunction, No. (%)	820 (64)	260/664 (39)	60/156 (38)	.87
RVSP, median (IQR), mm Hg	798 (63)	46 (37–54)	43 (34–55)	.16
RVSP ≥40 mm Hg, No. (%)	798 (63)	445/651 (68)	84/147 (57)	.009
Inferior Vena Cava Dilation, No. (%)	804 (63)	478/668 (72)	95/136 (70)	.68
Pericardial Effusion, moderate or greater, No. (%)	1,085 (85)	10/890 (1)	4/195 (2)	.29
Aortic Regurgitation, moderate or greater, No. (%)	1,276 (100)	17/1,040 (2)	8/236 (3)	.11
Aortic Stenosis, moderate or greater, No. (%)	1,276 (100)	10/1,040 (0.96)	9/236 (4)	.004
Mitral Regurgitation, moderate or greater, No. (%)	1,276 (100)	79/1,040 (8)	21/236 (9)	.50
Mitral Stenosis, moderate or greater, No. (%)	1,276 (100)	2/1,040 (0.19)	4/236 (2)	.01
Tricuspid Regurgitation, moderate or greater, No. (%)	1,276 (100)	194/1,040 (19)	50/236 (21)	.37

Abbreviations: AKI, acute kidney injury; ESRD, end-stage renal disease; IQR, interquartile range; LVEF, left ventricular ejection fraction; RVSP, right ventricular systolic pressure.

## Discussion

In this study, we present a large cohort of ICU patients from a 9-year period who had an echocardiogram performed within 7 days of CRRT initiation. Our group has previously described the incidence of certain echocardiographic abnormalities and their associated outcomes in ICU patients with septic shock [33, 34]. To our knowledge, the current study is the first to describe the prevalence of echocardiographic parameters in ICU patients receiving CRRT. We identified a high prevalence of echocardiographic abnormalities that differed by the type of ICU. Type of ICU admission is a surrogate for the actual condition that necessitated ICU-level care, and therefore, our findings describe and compare the echocardiographic characteristics between ICU patients on CRRT with different underlying pathologic processes. Echocardiographic parameters were also different between patients when assessed regarding the indication for CRRT (AKI vs. ESRD). We identified a high prevalence of LV diastolic dysfunction and a substantial prevalence of RV and LV systolic dysfunction. Markers of volume overload, including IVC dilation and pulmonary hypertension, were also common.

Diastolic dysfunction was common in our cohort (62%). According to a meta-analysis published in 2015, the prevalence of diastolic dysfunction in ICU patients with sepsis and septic shock is 48%, and it is associated with increased mortality risk [35]. However, individual studies have reported a prevalence of diastolic dysfunction that ranges from 20% to 57% [36, 37], and although its association with mortality has been previously reported, outcomes have not been consistent across the studies [36–38]. LV systolic dysfunction (defined as LVEF ≤45%) was noted in 32% of our patients; this rate is higher than that previously reported in a study that used even higher threshold values to define LV systolic dysfunction [38].

Prevalence of pulmonary hypertension (defined as RVSP ≥40 mm Hg) was also higher in our patient population (66%) compared with rates reported previously. In a study that used Doppler echocardiography to evaluate pulmonary hypertension (tricuspid regurgitant jet velocity ≥3 m/sec, which typically corresponds to RVSP ≥40 mm Hg), the prevalence of pulmonary hypertension in patients in a medical ICU was 42% [18]. Several possible mechanisms could account for the increased RVSP in ICU patients undergoing CRRT [39]. The most common cause of pulmonary hypertension in ICU patients is volume overload causing interstitial pulmonary edema and hypoxia-induced pulmonary artery vasoconstriction; left-sided heart failure is also common [39]. Invasive mechanical ventilation, which was used in 73% of our cohort, also may have contributed to increased RVSP. Parenchymal lung disease, another cause of pulmonary hypertension, was present in 16% of patients. RV systolic dysfunction (moderate or greater decrease in RV systolic function) was noted in 39%; this rate is slightly higher than the prevalence previously observed in ICU patients (28%-31%) [17, 38].

Tricuspid regurgitation (moderate or greater) was noted in 19% of patients; this rate is considerably higher than that previously reported (~3%) [40]. This association probably is attributable to the higher prevalence of pulmonary hypertension and RV systolic dysfunction in our cohort [41, 42].

The most common echocardiographic abnormality was IVC dilation (71%), consistent with volume overload in most patients who received CRRT. However, the majority of patients (73%) also were receiving mechanical ventilation, which can increase the diameter of the IVC [43]. Similarly, the substantial rates of RV systolic dysfunction, pulmonary hypertension, and tricuspid regurgitation may have contributed to IVC dilation. Notably, elevated central venous pressure, which may be reflected by a dilated IVC, is a known contributor to AKI because it is a component of the cardiorenal syndrome [44, 45].

Compared with patients in the noncardiac ICUs, patients admitted to the cardiac ICU more commonly had echocardiographic abnormalities, including diastolic dysfunction, IVC dilation, and valvular disease (aortic regurgitation and stenosis, tricuspid valve regurgitation). They also had a higher prevalence of LV systolic dysfunction and pulmonary hypertension. These findings are probably intrinsic to the cardiac causes that led to their admission to a cardiac ICU and possibly also to CRRT initiation (eg, cardiorenal syndrome).

Comparison of echocardiographic parameters between patients with AKI vs. ESRD showed a higher prevalence of LV systolic dysfunction (defined as LVEF ≤45%) in patients with ESRD, which likely reflects the high prevalence of cardiovascular disease and congestive heart failure in this patient population [46, 47]. In contrast, the prevalence of pulmonary hypertension (defined as RVSP ≥40 mm Hg) was higher in patients with AKI; high RVSP is a known causative factor of AKI because it indicates venous congestion and RV dysfunction (cardiorenal syndrome) [48, 49].

Our investigation has a number of important limitations inherent to its design as a retrospective, single-center, cross-sectional study; as a result, these findings may have limited generalizability. This study can only provide information regarding the association (not causation) between the prevalence of echocardiographic parameters and the different ICU patient populations (AKI vs. ESRD; type of ICU). Additionally, some of the echocardiographic parameters were assessed only in a small fraction of the patients including diastolic dysfunction (22% of patients) and left atrial volume index (19% of patients) which limits the internal validity of these parameters in our cohort. Furthermore, the indication for which each echocardiogram was performed was not available, and as a result, we could not assess the correlation of clinical indication with the prevalence of specific echocardiographic parameters.

Nevertheless, we report new information about a unique patient population that can be used to further investigate the association of echocardiographic parameters with adverse outcomes or modifiable risk factors in ICU patients receiving CRRT.

## Conclusions

We assessed a large cohort of ICU patients who received CRRT and noted an increased prevalence of pulmonary hypertension, diastolic dysfunction, and RV dysfunction. Patients in the cardiac ICU had higher prevalence rates for most echocardiographic abnormalities compared with patients admitted to noncardiac ICUs. Patients with AKI had a higher prevalence of pulmonary hypertension compared with patients with ESRD. Future studies that aim to predict adverse outcomes with echocardiographic parameters in this patient population can lead to the identification of modifiable risk factors and eventually improve patient care. These relationships must then be validated in prospective studies.

## Supporting information

S1 File De-identified dataset used for the analyses of this study. (XLSX)

## Abbreviations

AKI: acute kidney injury
APACHE III: Acute Physiology And Chronic Health Evaluation
CRRT: continuous renal replacement therapy
ESRD: end-stage renal disease
ICU: intensive care unit
IHD: intermittent hemodialysis
IQR: interquartile range
IVC: inferior vena cava
LV: left ventricle, left ventricular
LVEF: left ventricular ejection fraction
RV: right ventricle, right ventricular
RVSP: right ventricular systolic pressure
SOFA: Sequential Organ Failure Assessment
